# GOLPH3 inhibits glioma cell apoptosis through the JNK signaling pathway

**DOI:** 10.3389/fgene.2025.1518573

**Published:** 2025-01-29

**Authors:** Shao Xie, Jiahai Ding, Zhaohao Wang, Hengliang Shi, Zheng-Quan Yu

**Affiliations:** 1 Department of Neurosurgery, The First Affiliated Hospital of Soochow University, Suzhou, Jiangsu, China; 2 Department of Neurosurgery, The Affiliated Hospital of Xuzhou Medical University, Xuzhou, Jiangsu, China; 3 Department of Neurosurgery, Yantaishan Hospital Affiliated to Binzhou Medical University, Yantai, China; 4 Central Laboratory, The Affiliated Hospital of Xuzhou Medical University, Xuzhou, China

**Keywords:** glioma, GOLPH3, JNK, apoptosis, therapeutic target

## Abstract

**Background:**

Glioma, a primary intracranial tumor, is marked by high rates of mortality and disability, making it a significant health concern. Understanding the molecular mechanisms underlying glioma initiation and progression and identifying potential therapeutic targets for gene therapy are crucial for improving patient outcomes. Golgi phosphoprotein 3 (GOLPH3), predominantly localized at the trans-Golgi network, has been implicated in the pathogenesis of various cancers. However, its precise role in glioma progression remains under active investigation.

**Methods:**

To elucidate the function of GOLPH3, U87 glioma cells were transfected with GOLPH3-specific small interfering RNA (siRNA) to suppress its expression. An *in vivo* glioma model was generated by implanting GOLPH3-knockdown U87 cells into nude mice. Apoptosis was assessed using flow cytometry, immunofluorescence staining, TUNEL assays, and Western blotting. The activation of the JNK signaling pathway was evaluated by analyzing the phosphorylation levels of JNK and c-Jun through Western blotting.

**Results:**

Downregulation of GOLPH3 in U87 glioma cells significantly enhanced apoptosis, as evidenced by increased levels of cleaved caspase-3 and higher apoptosis rates. Furthermore, GOLPH3 knockdown led to the activation of the JNK signaling pathway, as indicated by elevated phosphorylation of JNK and c-Jun. *In vivo*, suppression of GOLPH3 expression inhibited tumor growth and increased apoptosis within the tumor microenvironment.

**Conclusion:**

These findings suggest that GOLPH3 might play a pivotal role in regulating apoptosis in malignant glioma cells via the JNK signaling pathway. Thus, GOLPH3 may represent a promising therapeutic target for glioma treatment.

## 1 Introduction

Glioma is a primary intracranial tumor with high mortality and disability rates, characterized by its invasion and unlimited proliferative ability (Huse et al., 2011; Schaff and Mellinghoff, 2023). Despite significant advancements in surgical techniques and adjuvant therapies, there has been minimal improvement in the median survival rates of affected patients over the past few decades (Rutka et al., 2000; Yu et al., 2009). Therefore, it is crucial to elucidate the potential mechanisms of glioma development and identify key molecular targets to develop more effective treatment methods.

Golgi phosphoprotein 3 (GOLPH3) is a key component of the trans Golgi apparatus network, playing a crucial role in the Golgi apparatus secretion pathway and protein glycosylation processes (Wu et al., 2000; Bell et al., 2001; Dippold et al., 2009; Tu et al., 2008; Schmitz et al., 2008). GOLPH3 has been identified as a novel oncogene in 2009 (Scott et al., 2009). Moreover, GOLPH3 is highly expressed in both the prostate and metastatic lymph nodes (Kielb et al., 2023). GOLPH3 is highly expressed in samples from NSCLC patients, and overexpression of GOLPH3 enhances metastasis and tumorigenicity by activating the WNT/β-catenin pathway (Song et al., 2021). GOLPH3 is overexpressed in colon cancer tissue and colon cancer cell lines. GOLPH3 promotes IL-6 induced STAT3 activation, followed by induction of integrin alpha 3 and ZEB1 transcription, thereby promoting the metastasis and progression of colon cancer (Huang et al., 2022). Subsequent studies emphasized the clinical significance of GOLPH3 in various cancers, including breast cancer, glioma, and other tumors. These studies consistently indicated that GOLPH3 was not only upregulated in these tumors, but also associated with poor prognosis and tumor development in these tumors (Hua et al., 2012; Li et al., 2012; Zhou et al., 2012; Zeng et al., 2012). Our previous research has shown that GOLPH3 is overexpressed in gliomas, and its downregulation hinders the proliferation of glioma cells *in vitro* and *in vivo* (Zhou et al., 2017). Specifically, GOLPH3 was found to promote glioma progression by inhibiting Rab5-mediated endocytosis and degradation of the epidermal growth factor receptor (EGFR) (Zhou et al., 2017). Ting Dai et al. found that GOLPH3 is a prognostic and/or potential therapeutic biomarker for hepatocellular carcinoma cell (HCC) patients, playing an important role in the activation of the NF-κB pathway during HCC progression (Dai et al., 2015). Activation of NF-κB signaling pathway can inhibit cell apoptosis and lead to the development of cancer (Van Antwerp et al., 1996). We have found that activation of the NF-κB signaling pathway can inhibit apoptosis of glioma cells (Xu et al., 2020; Guo et al., 2019), and the activation of NF-κB in gliomas can inhibit the activation of JNK signaling, thereby suppressing the apoptosis of glioma cells (Gupta et al., 2013; Srivastava et al., 2011). Nevertheless, the role of GOLPH3 in regulating glioma cell apoptosis, a fundamental characteristic of malignant glioma, remains to be elucidated.

In this study, we found that knockdown of GOLPH3 induced glioma cell apoptosis and elevated the activity of the JNK signaling pathway. These findings suggest that GOLPH3 plays a significant role in glioma pathogenesis and could serve as a potential therapeutic target for glioma treatment.

## 2 Materials and methods

### 2.1 Cell culture and reagent

U87 cell lines were purchased from the Chinese Academy of Sciences Shanghai Cell Bank. The cells were cultured in Dulbecco modified Eagle medium supplemented with 10% fetal bovine serum (Sijiqing Biotechnology Materials Co.) and F-12 (DMEM/F-12) (Gibco), and in a moist incubator at 37°C and 5% CO2. Before transfection with GOLPH3 siRNA, cells were treated with SP600125 (Sigma), a specific inhibitor of JNK, at a concentration of 10 μmol/L.

### 2.2 Antibodies

The antibodies used in this study are as follows:

mouse anti-c-Jun (sc-74543), rabbit anti-p-c-Jun (sc-16312), mouse anti-JNK (sc-7345), and mouse anti-p-JNK (sc-6254) (all from Santa Cruz Biotechnology); Rabbit anti-cleaved caspase-3 (#9661) and rabbit anti caspase-3 (#9662) (both from Cell Signaling); mouse anti-β-actin (Millipore, a5441); Rabbit anti-GOLPH3 (Abcam, ab236296); Rabbit anti-phospho-IKK (#2697), Rabbit anti-IKK (#8943), Rabbit anti-IκBα (#4812), Rabbit anti-phospho-IκBα (#2859) (all from Cell Signaling).

### 2.3 Small interfering RNA transfection and plasmids

Three specific siRNA duplexes (Shanghai Gene Pharma Co.) targeting human GOLPH3 were employed: GOLPH3-siRNA1 (5′-GAA​UUA​GCA​UUG​AGA​GGA​ATT-3′), GOLPH3-siRNA2 (5′-CAA​GAA​AGG​UAA​UCU​GUA​ATT-3′), and GOLPH3-siRNA3 (5′-GUU​AAG​AAA​UGU​ACG​GGA​ATT-3′). Each transfection was carried out in triplicate. Each transfection was carried out in triplicate. According to the manufacturer’s experimental protocol, we used Lipofectamine ™ 2000 (Invitrogen) transfection reagent was used for GOLPH3 siRNA transfection. The experimental cells were divided into two groups: knockout group (transfected with GOLPH3 siRNA oligonucleotide) and negative control group (transfected with negative control siRNA oligonucleotide). We seeded U87 cells into 6-well plastic culture plates, with approximately 3 × 105 cells per well. The culture medium is DMEM medium containing 10% without antibiotics. When the cells reach 60% fusion after 20 h, transfection can be performed. The dosage for each well is 10 µL siRNA and 5 µL liposomes, diluted with 250 µL Opti MEM. After standing at room temperature for 5 min, we mixed the two and incubated at room temperature for 20 min. Finally, we added the mixture directly into a 6-well plate. We gently shake and mix thoroughly. After culturing in the incubator for 5 h, we switched it to DMEM medium containing 10% calf serum for further cultivation. After 48 h, we collected transfected cells for testing.

The lentiviral core plasmid shGOLPH3 was constructed by our laboratory (Zhou et al., 2017). To established GOLPH3 knock-down U87 cells, we generated lentivirus based short hairpin RNA (shRNA) targeting human GOLPH3 with pLL3.7 as the backbone. Cells were infected with lentivirus containing GOLPH3 shRNA or a control scrambled short hairpin RNA (scramble). The shRNA sequences (5′-3′) targeting human GOLPH3 was listed below.

shGOLPH3: gtt​aag​aaa​tgt​acg​gga​att​caa​gag​att​ccc​gta​cat​ttc​tta​act​t ttttc (Forward);

tcg​aga​aaa​aag​tta​aga​aat​gta​cgg​gaa​tct​ctt​gaa​ttc​ccg​tac​att​tct​taa​ca (Reverse).

### 2.4 Flow cytometry analysis to examine cell apoptosis

To measure apoptotic cells, the membrane associated protein V-FITC apoptosis detection kit (Invitrogen) was used in this experiment. After treatment, glioma U87 cells were washed three times with frozen PBS and resuspended in buffer. Next, they were incubated in a dark environment for 15 min in FITC labeled Annexin V and PI, and evaluated using a flow cytometer.

### 2.5 Immunofluorescence

Immunofluorescence was conducted based on our previous study (Zhou et al., 2017). Cells were seeded on coverslips in 24 plates and fixed with 4% paraformaldehyde for 20 min after cells spreading. Next, the cells were permeabilized with 0.1% Triton X-100 for 10 min and subsequently blocked with 3% BSA for 30 min to minimize non-specific antibody interactions. The primary antibodies were incubated at 4°C overnight. Subsequent to incubation, cells were rinsed with PBS to eliminate any unbound antibodies. Afterwards, the cells were incubated for 2 h with a second antibody and DAPI was used to stain the nucleus. The cells were then observed utilizing laser scanning confocal microscopy (Olympus) for image capture. A rabbit anti-GOLPH3 antibody (ab236294) was added at 1:200, and the secondary antibody conjugated to Alexa Fluor 594 (1:500, Invitrogen) was employed to visualize the GOLPH3 staining in glioma cells. The cell nuclei were stained with 4,6-diamidino-2-phenyl- indole (DAPI; 1:1,000; Sigma).

### 2.6 Western blot analysis

According to the experimental design time, cells were lysed in RIPA Lysis and Extraction Buffer, and equal amounts of protein lysates were separated via 10% SDS-PAGE, and then transferred onto PVDF membranes (Millipore), and probed with primary antibodies (p-JNK, JNK, p-c-Jun, c-Jun, GOLPH3, cleaved caspase-3, caspase-3, phospho-IKK, IKK, Rabbit anti-IκBα, phospho-IκBα or β-actin) and incubated with horseradish peroxidase‐conjugated secondary antibodies. Detection was carried out using the Pierce ECL Plus Western blotting Substrate (Thermo Fisher Scientific Inc.).

### 2.7 Reverse transcription-polymerase chain reaction (RT-PCR)

Total RNA of the cells was extracted using the TRIzol Reagent (Tiangen Biotech Co.) and the cDNA was synthesized using reverse transcription reagents (Roche Applied Science) according to the instructions. The primer sequences were as follows.

β-actin: 5′-catgtacgttgctatccaggc-3′(forward); 5′-ctc​ctt​aat​gtc​acg​cac​gat-3′ (Reverse).

GOLPH3: 5′-tgtaagtcagatgctccaacagg-3′(forward); 5′-tcacccatttgtcaagaacgg-3′(Reverse).

### 2.8 Publicly available clinical data analysis

Data of GOLPH3 in TCGA were extracted from Betastasis (http://www.betastasis.com/), http://gepia.cancer-pku.cn/ and those in CGGA were extracted from http://www.cgga.org.cn/.

### 2.9 Intracranial glioma model in nude mice and brain slide preparation

The intracranial glioma of model nude mice was performed based on our previous study (Zhou et al., 2017). ShGOLPH3 or shNC U87 cells (1 × 10^6^) were injected into the right caudate nucleus of athymic nude mice (n = 6 per group). The tumor volume is calculated according to the formula V = 1/2 ab^2^, where “a” represents the longest diameter and “b” represents the shortest diameter. According to our preliminary research results, on the 20th day after implantation, all mice were anesthetized with chloral hydrate, perfused with 0.1 M PBS, and then perfused with 4% paraformaldehyde (PFA). Fresh frozen brains were sliced continuously with a thickness of 12 μm and stored at −80°C. All animal experiments were conducted in accordance with the guidelines for the care and use of experimental animals, and were approved by the Institutional Animal Care and Use Committee of Xuzhou Medical University.

Meanwhile, immunofluorescence technologies were used to evaluate tumors’ apoptosis by staining cleaved Caspase-3 and p-JNK. Slides were humidified for 15 min (3 times, 5 min each) at room temperature in PBS, permeabilized in 0.3% Triton X-100 in PBS for 30 min at room temperature and blocking for 1 h in 10% goat serum in wet box. Then following antibodies were used and overnight reacted in 4°C: rabbit anti-cleaved Caspase-3 antibody (1:200) and mouse anti-p-JNK antibody (1:200). In the next day, slides were incubated with the corresponding Alexa Fluor secondary antibodies (1:500; Invitrogen) for 1 h and DAPI (1:1,000; Sigma) for 10 min at room temperature. Microscopic images were obtained with an Olympus IX-71 fluorescence microscope. P-JNK and cleaved Caspase-3 positive cells were counted in three randomly selected high power fields.

### 2.10 TUNEL staining

The TUNEL staining procedure was measured according to the instructions of the TUNEL cell apoptosis detection kit (beyotime, catalog C1089). Slides were humidified for 15 min (3 times, 5 min each) at room temperature in PBS, permeabilized in 0.3% Triton X-100 in PBS for 30 min at room temperature. Fix the slices in a 4% methanol free formaldehyde solution in PBS. Subsequently, 50 μL of TUNEL detection solution (including 5 μL of TdT enzyme and 45 μL of fluorescent labeling solution) was added to each slice and incubated at 37°C in the dark for 60 min. DAPI is used as a nuclear staining agent for cell visualization. Identify apoptotic cells using fluorescence microscopy.

### 2.11 Statistical analysis

In this study, we used SPSS 17.0 software for statistical analysis. All experimental data were conducted in triplicate, and all result data were shown as mean ± SD. Student’s t-test was used to statistically analyze between different groups. After statistics, if the p-value is less than 0.05, we consider it to be statistically significant, *p < 0.05, **p < 0.01.

## 3 Results

**FIGURE 1:**
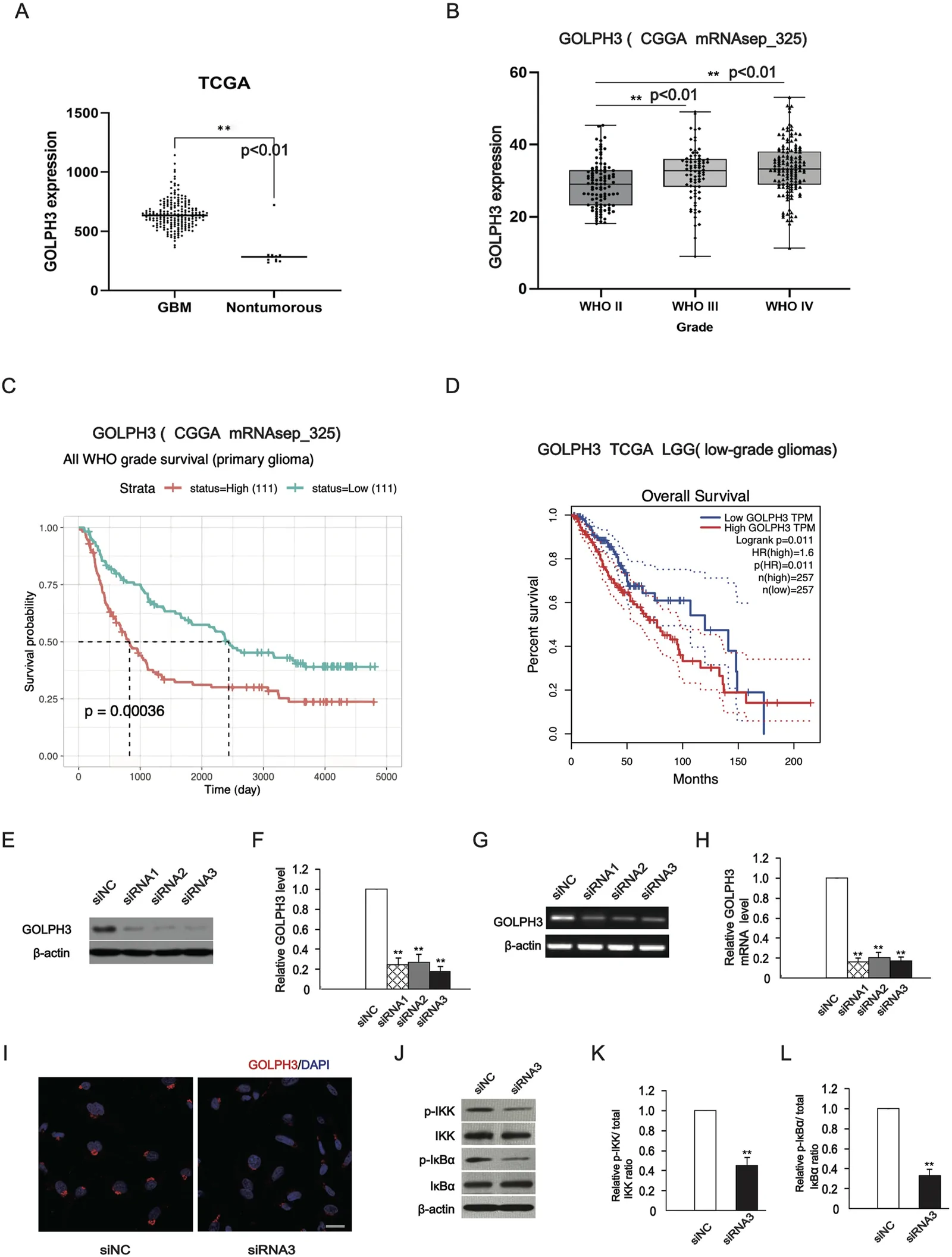
Validation of downregulation of GOLPH3 in U87 glioma cells. **(A, B)** According to TCGA and CGGA, the expression of GOLPH3 in glioma tissue. **(C, D)** The expression level of GOLPH3 in the CGGA dataset is negatively correlated with patient’s survival possibility, and gliomas with a high GOLPH3 level exhibited shorter survival time in the TCGA dataset. The high and low expression of GOLPH3 are grouped according to the median value of the expression of GOLPH3 gene, with those above the median defined as high expression and those below the median defined as low expression. **(E, F)** The representative images (immunoblotting) showed that the downregulation effect of GOLPH3 exceeded 70%, and statistical results of protein expression in cells after transfection. **(G)** RT-PCR method was used to detect GOLPH3 mRNA expression levels in cells transfected with small interference group (GOLPH3-siRNA 792, siRNA 635 and siRNA 571) and negative control group (NC siRNA) for 48 h. **(H)** Statistical results of mRNA expression in cells after transfection. **(I)** A typical immunofluorescence staining image shows that the specific subcellular location of endogenous GOLPH3 is located around the nucleus. After downregulation of GOLPH3, the fluorescence staining intensity decreased. Scale bar: 20 μm. **(J–L)** Western blotting analysis of IKK, p-IKK, IκBα and p-IκBα expression in the U87 cells and statistical graphs. The control group was normalized to 1, and the relative values of the other groups were calculated based on the control group. The quantitative statistical chart shows the relative ratio and relative activity.

The increase in GOLPH3 protein levels in glioma tissues, was confirmed in our previous research (Zhou et al., 2017). In addition, we found significant upregulation of GOLPH3 mRNA levels in gliomas through analysis of TCGA and CGGA databases (Figures 1A, B) and patients with high levels of GOLPH3 expression have a poorer prognosis (Figures 1C, D). We used RNA interference to downregulate the expression of GOLPH3. Western blot and RT-PCR analysis confirmed that GOLPH3 was effectively knocked down with all three siRNA duplexes (Figures 1E–H). We also observed a decrease in the fluorescence intensity of GOLPH3 staining around the nucleus after downregulation with siRNA using immunofluorescence staining (Figure 1I). Finally, we observed that the levels of phosphorylated-IKK and -IκBα were significantly decreased (Figures 1J–L) after downregulation with siRNA, indicating that downregulating of GOLPH3 inhibits the activation of the NF-κB signaling pathway.

**FIGURE 2:**
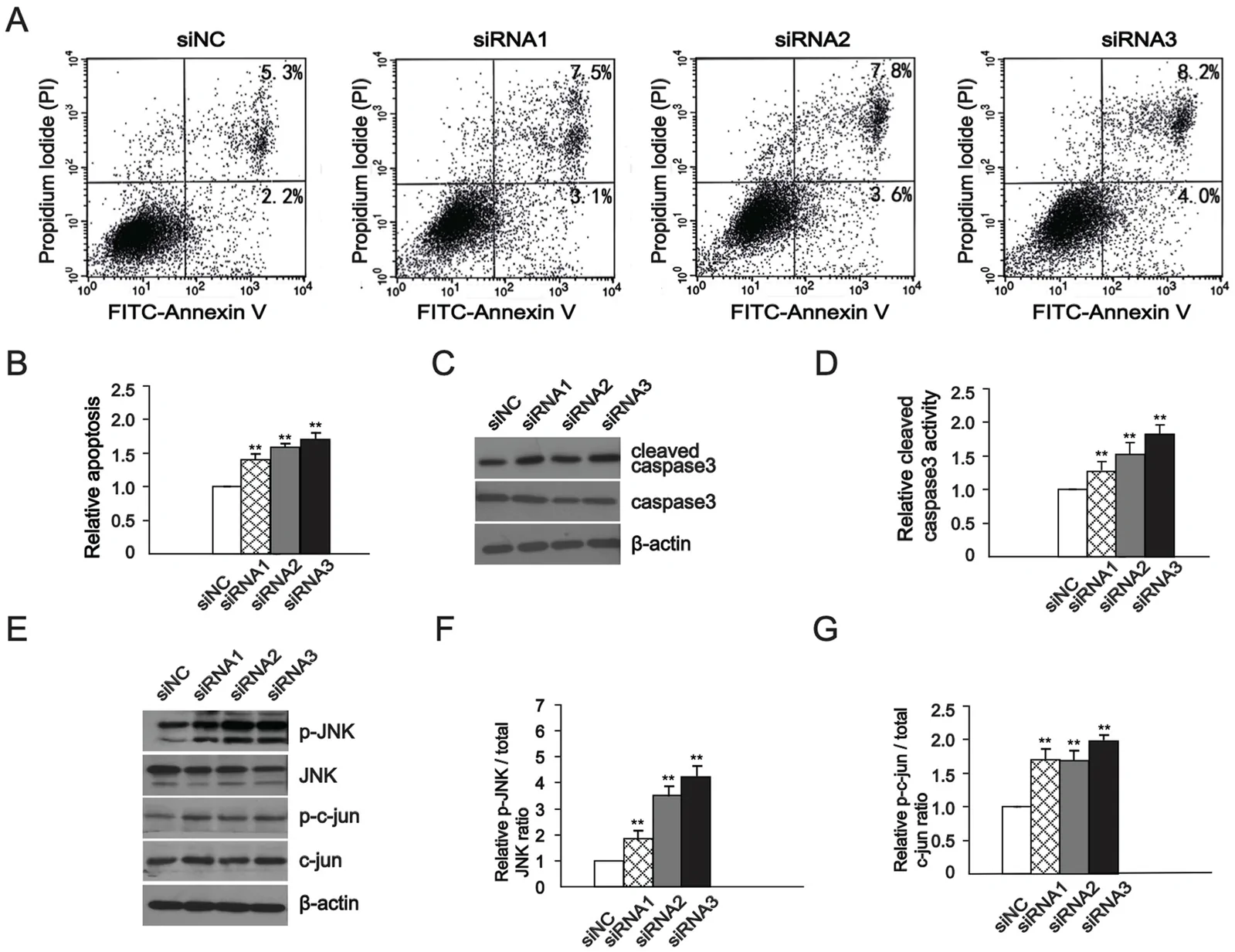
GOLPH3 regulated glioma cell apoptosis through the JNK signaling pathway, **(A, B)** After siRNAs transfection, Flow cytometry detection of cell apoptosis rate and quantitative analysis of relative apoptosis rate. **(C, D)** Quantitative analysis of protein level of cleaved caspase 3 and caspase 3 changes in WB experiment and statistical graphs. **(E–G)** Western blot analysis of protein levels of JNK, phospho-JNK, c-Jun, and phospho-c-Jun and statistical graphs, and the elevated expression is a result of GOLPH3 downregulation. The control group was normalized to 1, and the relative values of the other groups were calculated based on the control group. The quantitative statistical chart shows the relative apoptosis rate, ratio, and relative activity.

We used flow cytometry to detect the effect of downregulation of GOLPH3 expression on apoptosis in U87 glioma cell. After 48 h of transfection with the same technology, the average apoptosis rates of U87 cells detected by flow cytometry were 10.27%, 11.64%, and 12.49%, respectively (Figure 2A), compared to siNC (7.35%). These numbers are the mean of apoptosis rates. Quantitative analysis of apoptotic cells showed a significant increase in apoptosis relative to the siNC group (Figure 2B). In addition, we also detected the effect of downregulation of GOLPH3 on cleaved caspase-3, a key cell apoptosis activator and effector (Elmore, 2007). The levels of cleaved caspase-3, the active form of caspase-3, significantly increased following GOLPH3 downregulation compared with the control group (Figures 2C, D). Based on the above experimental results, it is indicated that downregulating GOLPH3 promoted apoptosis of glioma cells.

**FIGURE 3:**
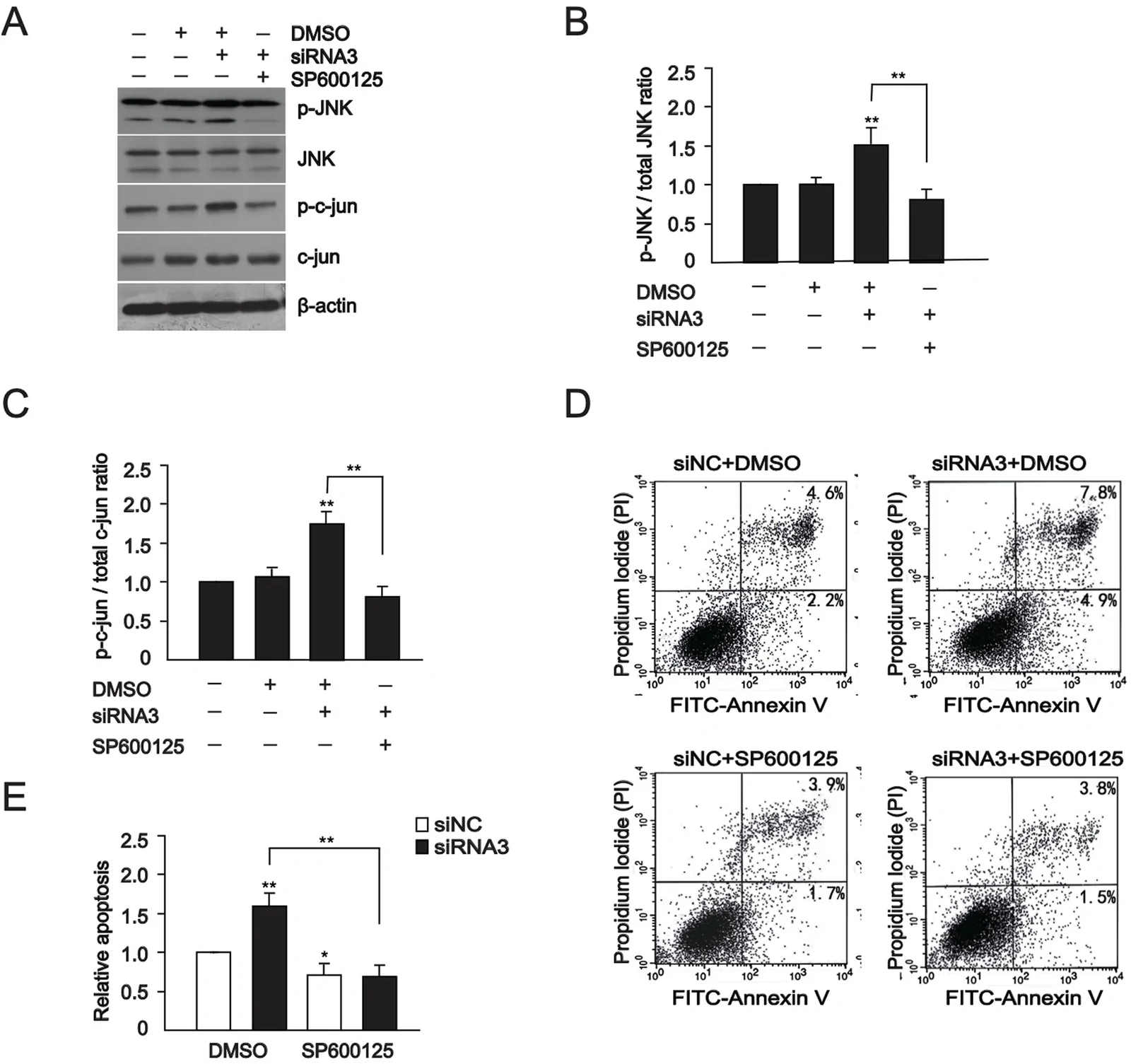
The increased JNK signaling is eliminated by JNK inhibitor. **(A)** Western blot analysis of protein levels of JNK, phospho-JNK, c-Jun, and phospho-c-Jun at 48 h after transfection with GOLPH3 siRNA3 with or without pretreatment with 10 μM SP600125 for 1 h **(B, C)** Quantitative analysis of JNK, phospho-JNK, c-Jun, and phospho-c-Jun in U87 cells. **(D, E)** Apoptosis of cells was assessed by flow cytometry analyses of annexin V-FITC/PI double-staining at 48 h after transfection with GOLPH3 siRNA3 with or without pretreatment with 10 μM SP600125 for 1 h, and statistical graphs. The control group was normalized to 1, and the relative values of the other groups were calculated based on the control group. The quantitative statistical chart shows the relative apoptosis rate, ratio, and relative activity.

The activation of JNK mediated signaling pathways can directly enhance the activity of transcription factors such as c-Jun in the nucleus and activated the c-Jun-c-Fos complex AP-1. After transcriptional activation, AP1 further upregulates the expression of apoptotic proteins, promoting cell apoptosis (Ho et al., 2024; Zhang et al., 2024; Dhanasekaran and Reddy, 2008). In the following experiment, we observed the impact of GOLPH3 downregulation on the JNK signaling. We found that levels of JNK and c-Jun protein levels increased in the U87 cells (Figures 2E–G), indicating that the JNK signaling pathway is either directly or indirectly regulated by GOLPH3 in glioma cells. To further verify whether GOLPH3 influences cell apoptosis through the JNK pathway, we used a JNK inhibitor, SP600125, to evaluate whether it can reverse the cell apoptosis induced by GOLPH3 (Hetschko et al., 2008). Cells were transfected with GOLPH3 siRNA3 for 48 h, with or without pretreatment with 10 μM SP600125 for 1 h. Western blot showed that the increased levels of phosphorylated JNK and c-Jun induced by downregulating GOLPH3 decreased after treatment with the JNK inhibitor SP600125 (Figures 3A–C). In addition, flow cytometry assay exhibited that the pro-apoptotic effect of downregulating GOLPH3 was reversed by the addition of JNK inhibitor SP600125 (Figures 3D, E). These data demonstrated that GOLPH3 may inhibit cell apoptosis through the JNK signaling.

**FIGURE 4:**
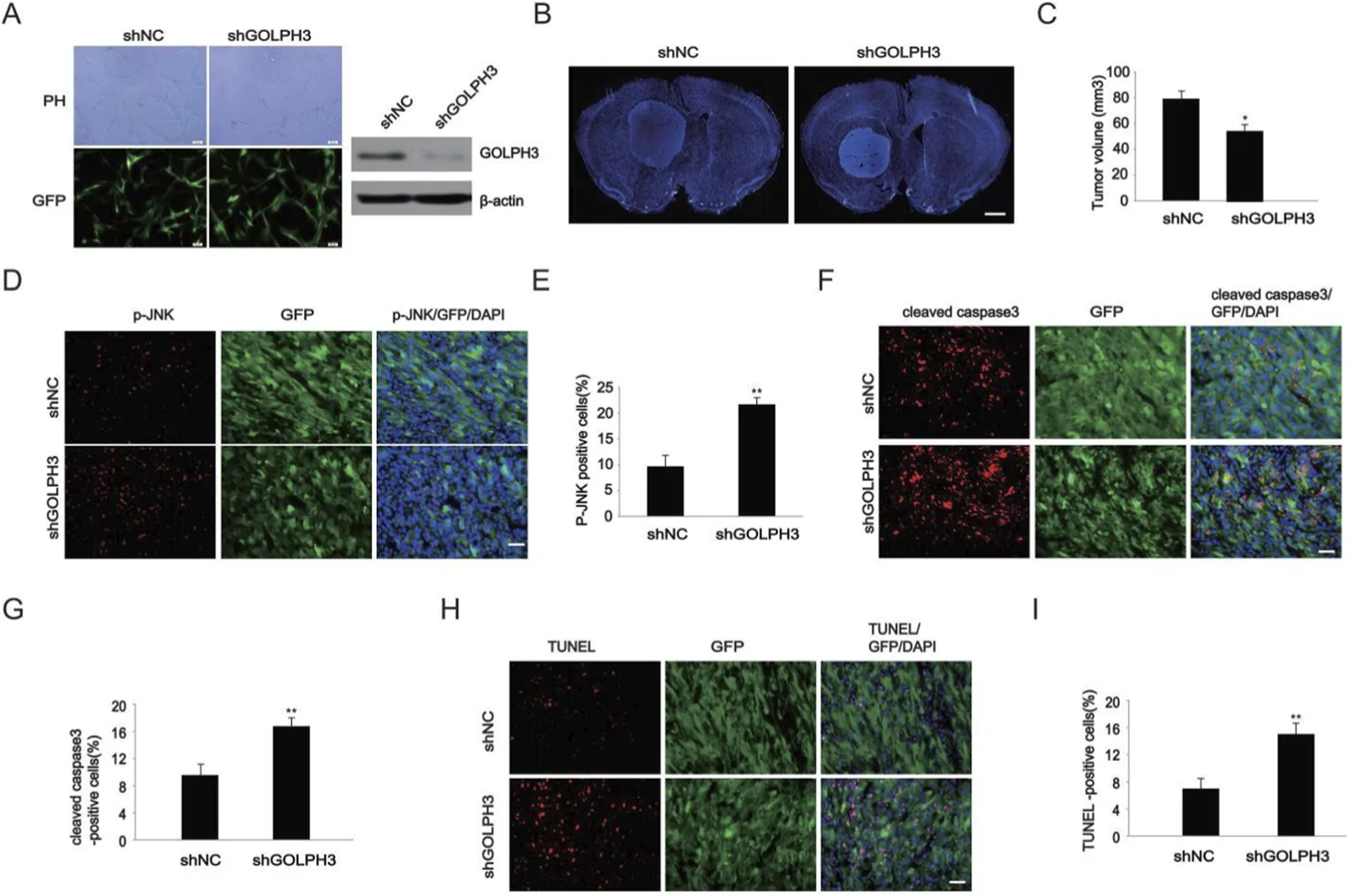
Detection of the effect of GOLPH3 on glioma apoptosis *in vivo*. **(A)** Construction of U87 glioma cells stably down-regulating GOLPH3. The proportion of lentiviral infection and the effect after infection are identified using fluorescence and WB experiment. The representative image (left) and immunoblot (right) show that the downregulation of GOLPH3 has a therapeutic effect of over 80%. Scale bar: 100 μm. **(B)** DAPI fluorescence staining was performed using brain slices of intracranial gliomas. Scale bar: 1 mm. **(C)** Quantitative analysis of the volume of intracranial gliomas. **(D)** Representative images of p-JNK staining in tumor brain tissue derived from shNC and shGOLPH3 cells. Scale bar: 20 um. **(E)** Quantitative analysis of the p-JNK positive cells. **(F)** Representative images of cleaved caspase 3 staining in tumor brain tissue derived from shNC and shGOLPH3 cells. Scale bar: 20 um. **(G)** Quantitative analysis of the cleaved caspase 3 positive cells. **(H)** Representative images of TUNEL staining in tumor brain tissue derived from shNC and shGOLPH3 cells. Scale bar: 20 um. **(I)** Quantitative analysis of the TUNEL positive cells.

To investigate the effect of GOLPH3 on cell apoptosis in nude mice *in vivo*, we generated stably downregulating GOLPH3 U87 cells through the lentiviral system. As shown in Figure 4A, based on the percentage of GFP positive cells and Western blot results, the downregulation efficiency of GOLPH3 was validated. Next, we used stereotactic technology to transplant the U87 cells into the right striatum of nude mice to establish an intracranial glioma model. As shown in Figure 4B, DAPI staining revealed tumor formation in the right striatum, with a higher cell density than normal brain tissue. Meanwhile, tumors derived from cells with downregulation of GOLPH3 were smaller than those derived from control cells (Figures 4B, C). These finding indicated that downregulation of GOLPH3 inhibited glioma growth *in vivo*.

Next, we performed immunofluorescence staining on mouse brain slices and found that the percentage of p-JNK positive cells in tumors from the shGOLPH3 group was 21.77%, which was higher than the control group (9.68%) (Figures 4D, E). Cleaved caspase-3 is the most used biomarker for cell apoptosis. We found that the percentage of cleaved caspase-3 positive cells from the shGOLPH3 group was 16.77%, which was higher than the percentage of cells from the control group (9.6%) (Figures 4F, G). Finally, we performed TUNEL fluorescence staining on mouse brain slices and found that the percentage of TUNEL positive cells in tumors derived from GOLPH3 downregulated cells was 14.99%, which was higher than the 6.99% in the control group (Figures 4H, I). These data indicate that downregulation of GOLPH3 promotes tumor cell apoptosis.

## 4 Discussion

In our experimental study, we first found that GOLPH3 affects glioma cell apoptosis and the JNK signaling pathway was either directly or indirectly regulated by GOLPH3 in glioma cells. Since 2009, Scott et al. first confirmed that GOLPH3 is an oncogene in Nature magazine (Scott et al., 2009), it has been confirmed to be highly expressed in colorectal cancer, lung cancer, breast cancer, and advanced adenocarcinoma, correlating with the occurrence and development of tumors (Hua et al., 2012; Li et al., 2012; Zhou et al., 2012; Zeng et al., 2012). Recent studies have further emphasized that GOLPH3 is highly expressed in human gliomas and affects the proliferation, invasion, and prognosis of gliomas (Zhou et al., 2012; Zhou et al., 2013; Li et al., 2011). However, the role played by GOLPH3 in glioma cell apoptosis and the mechanisms by which it affects cell apoptosis are still unclear.

Malignant gliomas endanger the lives of patients. Although surgery, radiation therapy, and chemotherapy are used, the survival rate is still low. The main reasons are the infinite proliferation of glioma cells, their strong invasiveness, and reduced cell apoptosis (Li et al., 2011; Gao et al., 2024). Our previous research indicated that GOLPH3 is upregulated in gliomas (Zhou et al., 2017). In our study, using flow cytometry detection and WB experiments, we found that downregulating GOLPH3 increased the apoptosis rate of glioma u87 cells, as well as increased the expression of cleaved caspase-3, which plays a crucial role in cell apoptosis. We have known that GOLPH3 plays an important role in the activation of the NF-κ B pathway during the progression of HCC (Dai et al., 2015). However, the relationship between GOLPH3 and NF-κB in glioma cells is still unclear. We have confirmed that knockdown of GOLPH3 inhibits the activation of the NF-κB pathway in glioma cells and the relationship between GOLPH3 and NF-κB pathway is consistent in glioma and HCC.

JNKs, part of the mitogen-activated protein kinases (MAPKs) family, are implicated in various cellular processes, including proliferation, apoptosis, and DNA damage repair (Li et al., 2013; Tomicic et al., 2015; Shi et al., 2023). JNK signals can promote cell growth upon acute stimulation, whereas chronic stimuli typically induce apoptosis (Ho et al., 2024). Following chronic activation, JNK is phosphorylated and activated, which subsequently regulates expression of apoptosis-related genes like FasL and TNF (Gao et al., 2024; Li et al., 2013; Schepetkin et al., 2023). Literature indicates that activation of the JNK signaling pathway promotes apoptosis in glioma cells (Tomicic et al., 2015; Shi et al., 2023). Our study indicated that downregulating of GOLPH3 in glioma U87 cells activated JNK phosphorylation and downstream signal expression, and the activated signal can be reversed by SP600125. At the same time, we verified that knockdown of GOLPH3 inhibited apoptosis *in vivo*, as reflected by the increases in the phosphorylation of JNK, the cleaved caspase3 and the TUNEL-positive cell rate. We believe that the JNK signaling pathway is either directly or indirectly regulated by GOLPH3 in glioma cells. The discovery of this mechanism can provide a theoretical basis for the use of JNK agonists as anti-tumor drugs in glioma patients with high expression of GOLPH3. This personalized treatment brings new hope to glioma patients, and we will continue to improve drug experiments in future.

## 5 Conclusion

We conclude that GOLPH3 inhibits glioma cell apoptosis and the JNK signaling pathway is either directly or indirectly regulated by GOLPH3 in glioma cells. The discovery of this mechanism can provide a theoretical basis for the patients with high expression of GOLPH3. We hope that our findings can provide new insights for personalized targeted therapy of glioma patients.

## Data Availability

The original contributions presented in the study are publicly available. This data can be found here: https://figshare.com/s/9e156db3d40ceab42715.
