# Correction: GOLPH3 inhibits glioma cell apoptosis through the JNK signaling pathway

**DOI:** 10.3389/fgene.2026.1943337

**Published:** 2026-10-05

**Authors:** Shao Xie, Jiahai Ding, Zhaohao Wang, Hengliang Shi, Zheng-Quan Yu

**Affiliations:** 1 Department of Neurosurgery, The First Affiliated Hospital of Soochow University, Suzhou, Jiangsu, China; 2 Department of Neurosurgery, The Affiliated Hospital of Xuzhou Medical University, Xuzhou, Jiangsu, China; 3 Department of Neurosurgery, Yantaishan Hospital Affiliated to Binzhou Medical University, Yantai, China; 4 Central Laboratory, The Affiliated Hospital of Xuzhou Medical University, Xuzhou, China

**Keywords:** glioma, GOLPH3, JNK, apoptosis, therapeutic target

There was a mistake in Figure 4D as published. The p-JNK, GFP, and p-JNK+GFP+DAPI merged images for the shNC group were inadvertently replaced with incorrect source files during figure compilation. The corrected Figure 4D appears below.

**FIGURE 4:**
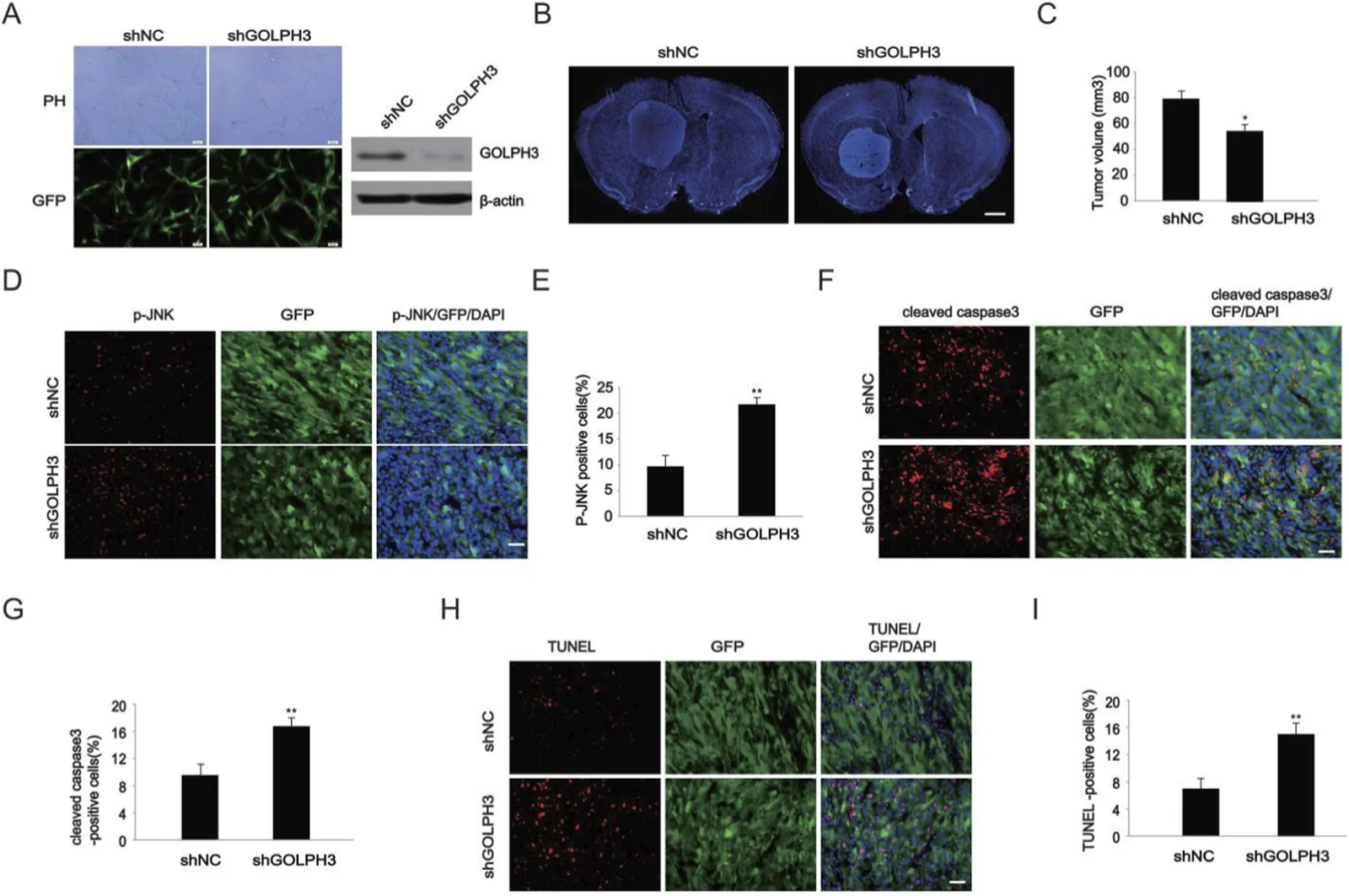
Detection of the effect of GOLPH3 on glioma apoptosis *in vivo*. **(A)** Construction of U87 glioma cells stably down-regulating GOLPH3. The proportion of lentiviral infection and the effect after infection are identified using fluorescence and WB experiment. The representative image (left) and immunoblot (right) show that the downregulation of GOLPH3 has a therapeutic effect of over 80%. Scale bar: 100 μm. **(B)** DAPI fluorescence staining was performed using brain slices of intracranial gliomas. Scale bar: 1 mm. **(C)** Quantitative analysis of the volume of intracranial gliomas. **(D)** Representative images of p-JNK staining in tumor brain tissue derived from shNC and shGOLPH3 cells. Scale bar: 20 um. **(E)** Quantitative analysis of the p-JNK positive cells. **(F)** Representative images of cleaved caspase 3 staining in tumor brain tissue derived from shNC and shGOLPH3 cells. Scale bar: 20 um. **(G)** Quantitative analysis of the cleaved caspase 3 positive cells. **(H)** Representative images of TUNEL staining in tumor brain tissue derived from shNC and shGOLPH3 cells. Scale bar: 20 um. **(I)** Quantitative analysis of the TUNEL positive cells.

The original version of this article has been updated.

